# Reconstruction of mechanical leg axis using non-modular cemented hinged prosthesis in complex primary total knee arthroplasty

**DOI:** 10.1007/s00402-024-05409-z

**Published:** 2024-07-11

**Authors:** Benjamin Jacob, Nadja Jacob, Eric Röhner, Georgi Wassilew, Georg Matziolis, Markus Heinecke

**Affiliations:** 1 Orthopaedic Department Waldkliniken Eisenberg, University Hospital Jena, Campus Eisenberg, Eisenberg, Germany; 2 Orthopaedic Department of the Heinrich-Braun-Hospital Zwickau, Campus Eisenberg, Klosterlausnitzer Straße 81, 07607 Eisenberg, Germany; 3 Department of Orthopaedic Surgery, University Hospital Greifswald, Greifswald, Germany

**Keywords:** Knee arthroplasty, Modularity, Mechanical axis, Rotating hinged knee, Periprosthetic fracture

## Abstract

**Purpose:**

Modular cementless knee arthroplasty systems are capable of precise reconstruction of the mechanical axis. However, they are considered more susceptible to complications. In contrast, non-modular cemented systems are said to be more forgiving and show good long-term results. The aim of this study was to investigate the resulting leg axis after implantation of a non-modular cemented rotating hinged knee prosthesis. Furthermore, potential risk factors for the occurrence of malalignment and complications should be identified.

**Methods:**

Between 2005 and 2015, 115 patients could be included in this monocentric retrospective cohort study. All patients underwent primary hinged non-modular cemented total knee arthroplasty. Preoperative and postoperative standardized long radiographs were analysed to determine resulting leg axis. Furthermore, epidemiological and intraoperative data as well as perioperative complications were surveyed.

**Results:**

Average leg axis was 5.8° varus preoperatively and 0.6° valgus postoperatively. Considering an axis deviation of 3° as the target corridor, 27% of all cases examined were outside the desired range. 21% cases showed a femoral deviation from the target corridor and 15% showed a tibial deviation. There was a significant relationship between the preoperative mLDFA and the mechanical alignment of the femoral component (R = 0.396, p < 0.001) as well as between the preoperative mMPTA and the mechanical alignment of the tibial component (R = 0.187, p = 0.045). The mean operative duration was 96 min. No periprosthetic fractures were observed within the study cohort.

**Conclusion:**

The main result of the present work is that a non-modular cemented rotating hinged knee arthroplasty system can reconstruct the mechanical leg axis precisely and comparable to modular cementless and unconstrained total knee prostheses. Component malalignment is primarily dependent upon extraarticular deformity preoperatively. Periprosthetic fracture rates and duration of surgery were lower compared with current literature.

**Level of evidence:**

Level III: Retrospective cohort study.

## Introduction

The use of hinged systems in primary total knee arthroplasty (TKA) remains infrequent, numbering 0.2–2.8% in major national arthroplasty registries [2, 27, 30, 31]. Complication rates in the range of 9.2–63% and 10-year survivorship of 51–92.5% were reported [5]. However, if considering the selection bias in publications where rotating hinged knees (RHK) are merely used in disastrous cases as “salvage” implants, recent studies have confirmed reasonable survival rates and function [1, 8, 9, 16, 18, 21]. Nevertheless, indications for a RHK in a primary setting should be limited to collateral ligament insufficiency, severe varus or valgus deformity (> 20°), extensive flexion–extension gap imbalance, ankylosis and hyperlaxity [13].

In recent years modular cementless knee arthroplasty systems are becoming increasingly popular due to their ability to address individual anatomy, allowing precise reconstruction of the mechanical axis [30]. However, relevant complications (i.e. periprosthetic fractures, postoperative implant migration, end-of-stem pain) have been reported [10, 11, 19, 26]. Furthermore, fully integrated coated implants can be challenging to revise.

In contrast, cemented non-modular systems are simpler to implant due to their lack of complexity. While this often results in the stem not being able to be implanted parallel to the tibial or femoral diaphysis, this can be compensated for by the cement mantle to a certain amount.

In this work, the resulting leg axis after primary hinged non-modular cemented total knee arthroplasty was investigated. Furthermore, potential risk factors for the occurrence of a malalignment and periprosthetic fractures were identified. We hypothesized that reconstruction of the mechanical leg axis is precisely possible and that modularity is unnecessary for the majority of cases.

## Materials and methods

**Fig. 1:**
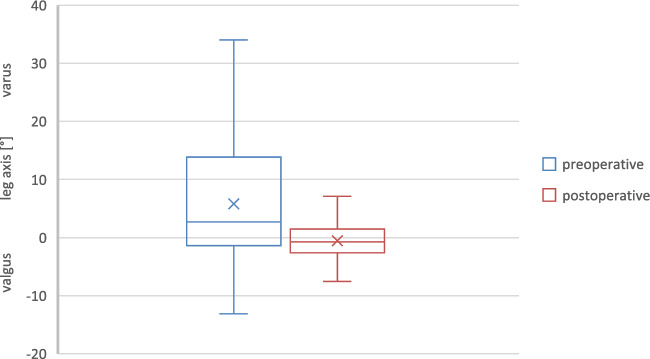
Box plot of preoperative and postoperative mechanical leg axis [°]

This is a monocentric retrospective cohort study. It was approved by the local ethics committee (2023–3183). Resulting from the retrospective and anonymized study design, an informed consent was not necessary according to the ethics committee approval. All methods were carried out in accordance with the relevant guidelines and regulations based on the approval. Between 2005 and 2015 all patients receiving a primary non-modular cemented rotating hinged knee arthroplasty (Endo Model, Waldemar Link, Hamburg, Germany) for the treatment of osteoarthritis of the knee were included (Fig. 1a–d). Indications for using a primary RHK were severe varus or valgus deformity (> 20°), extensive flexion–extension gap imbalance, ankylosis and hyperlaxity. Exclusion criteria were revision cases.

Epidemiological data (sex, age, BMI), intraoperative data (duration of surgery, side) and perioperative complications (periprosthetic fractures) were registered. Radiographic data was collected pre- and postoperatively before discharge (five days after surgery) by two independent blinded evaluators. The leg axis was determined on standardized whole leg standing radiographs. The amount of preoperative instability was measured as the joint line convergence angle (JLCA) between the tangent line of the femoral condyle and the tibial plateau. After DICOM files were imported to ImageJ (https://imagej.nih.gov/ij) angles were calculated based on the following points, which were registered consecutively: hip centre, knee centre, ankle centre, lateral and medial femoral condyle, lateral and medial tibial plateau [14]. All points were registered with an accuracy better than 1 mm. The angles were calculated (Microsoft Excel, Microsoft Corporation, Redmont, Washington, USA) using standard trigonometry, so that the resulting values should have an accuracy better than 1°. The inaccuracy of long standing radiographs regarding rotation and flexion of the leg was addressed by performing the radiographs in a standardized manner. The radiographs were taken in full extension or max. 5° of flexion and immediately checked for correct rotation and repeated if necessary. Correct rotation was defined as central projection of the femoral trochlea in reference to the condyles of the femoral implant.

### Statistical analysis

Statistical analysis was performed using SPSS (Ver. 24, IBM). After testing for normal distribution using the Kolmogorov–Smirnov–test all data are given as mean values ± standard deviation. The Pearson correlation coefficient was calculated for the preoperative mLDFA and mMPTA and the postoperative alignment of the femoral and tibial component. For all tests a p-value of < 0.05 was considered as statistically significant.”

## Results

Over 11 years, 115 patients were included in the study (41 male, 74 female). Medium age at time of surgery was 68.5 years, average BMI 30.4 kg/m^2^ (Table 1). Mean duration of surgical intervention was 96 min (Table 1). No periprosthetic fractures were detected neither intraoperatively nor postoperatively until discharge.

**Table 1:** Epidemiological, intraoperative and radiographic data

**Epidemiological data**	
Number	115
Male	41
Female	74
Age (years)	68.5 ± 11
BMI (kg/m^2^)	30.4 ± 6
**Intraoperative data**	
Duration of surgery (min.)	96 ± 41
Right side (cases)	62/115
Left side (cases)	53/115
Periprosthetic fractures	0/115
**Radiographic data—preoperative**	
Mechanical leg axis	5.80° varus
Instability (JLCA)	8.02° varus
**Radiographic data—postoperative**	
Mechanical leg axis	0.55° valgus
Deviation	2.5° ± 2.1°
> 3° target corridor (cases)	31/115
	11/115 varus
	20/115 valgus
	24/115 femoral
	17/115 tibial

**Fig. 2:**
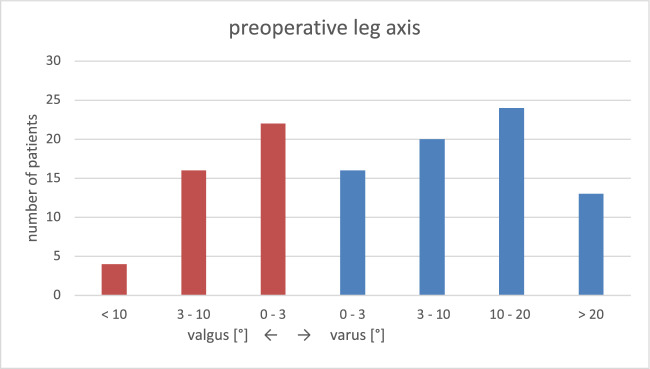
Bar chart of preoperative mechanical leg axis [°]

**Fig. 3:**
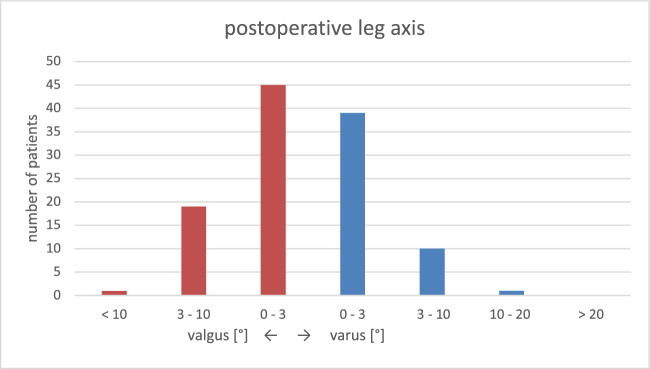
Bar chart of postoperative mechanical leg axis [°]

**Fig. 4:**
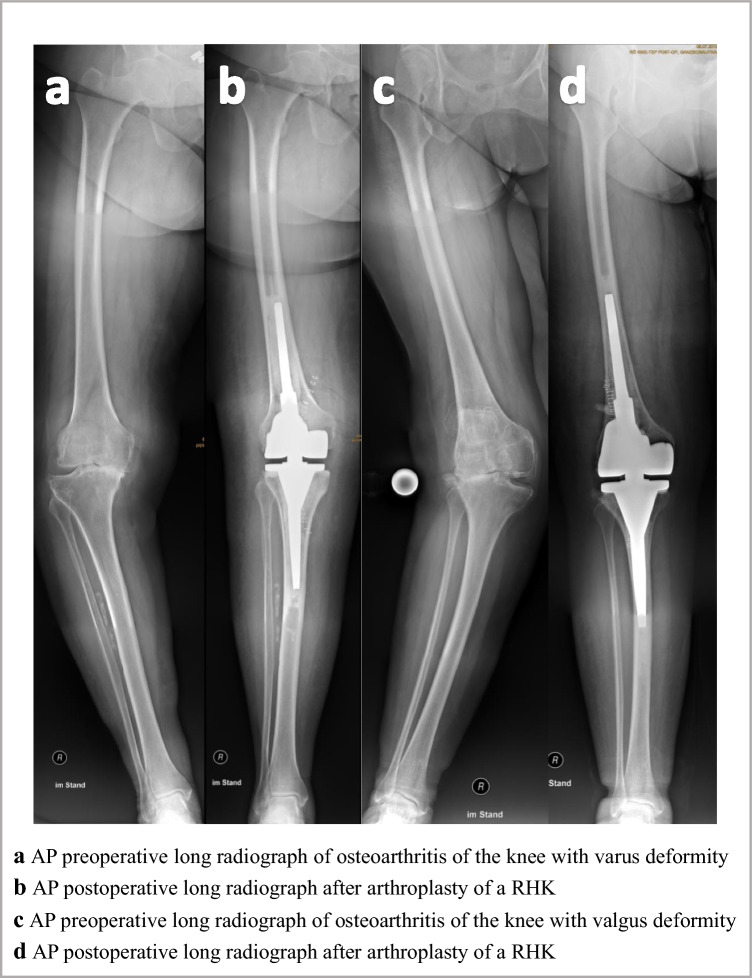
Images **a** AP preoperative long radiograph of osteoarthritis of the knee with varus deformity. ** b** AP postoperative long radiograph after arthroplasty of a RHK. **c** AP preoperative long radiograph of osteoarthritis of the knee with valgus deformity. **d** AP postoperative long radiograph after arthroplasty of a RHK

The average preoperative leg axis was 5.8° varus with instability (JLCA) of 8.0° varus (Table 1, Figs. 2 and 3). The average postoperative leg axis was 0.6° valgus with deviation from the mechanical axis of 2.5° ± 2.1° (0°–10.3°) (Figs. 2, 3, 4). Considering three degrees of deviation as the target corridor, 27% of all cases examined were outside the desired range (31/115; 11 varus, 20 valgus) (Fig. 4). Femoral deviation from the target corridor was seen in 21% (24/115) and tibial deviation in 15% (17/115) of all cases observed (Table 1).

We calculated a significant relationship between the mLDFA and the mechanical alignment of the femoral component (R = 0.396; p < 0.001) as well as the mMPTA and the mechanical alignment of the tibial component (R = 0.187; p = 0.045), i.e. the preoperative femoral/tibial deformity or extraarticular defect leads to a postoperative component malpositioning in the same direction.

## Discussion

The main result of the present work is that a non-modular cemented rotating hinged system can reconstruct the mechanical leg axis to the same extent as modular systems or conventionally instrumented unconstrained total knee prostheses. To our knowledge this is the first study to examine mechanical leg alignment after primary RHK in such a large cohort.

In our study 27% of all cases examined were outside the 3° target corridor. Cheng et al., Mason et al. and Fu et al. reported outliers of 28.3%, 31.8 and 28.7% in their meta-analyses considering conventional total knee arthroplasty [7, 12, 20].

Our surveyed group exhibited a mean absolute coronar deviation from the neutral mechanical leg axis of 2.5° ± 2.1° postoperatively. These results are comparable to those of Ochs et al. who reported a deviation of 2.7° ± 1.1° postoperatively for primarily implanted hinged arthroplasties. They were using computer-assisted navigation along with a modular rotating-hinge knee arthroplasty system (EndoRo, Aesculap AG, Tuttlingen, Germany) and cementless stem fixation in some of their patients [22]. Boelch et al. compared RHK designs (Link Endo-Model SL vs. EnduRo) for revision knee arthroplasty in patients with gross ligament instability and concluded that “the Endo-Model yielded straighter legs” [4]. The authors tried to attribute their results to the significantly longer cemented stems of this specific non-modular implant.

Stem fixation techniques are controversially discussed in the literature. While some authors published comparable survivorship of cementless press-fit stems, others reported increased micromotion and higher incidence of end-of-stem pain [10, 11, 29]. Additionally cemented stems allow more variability of component positioning without modularity. This prevents lateral oversizing and gives the possibility to reconstruct mechanical leg axis precisely even when an implantation not parallel to the tibial or femoral diaphysis is required (i.e. moderate extraarticular deformities). With cementless press-fit stems this can only be achieved by off-set adapters which increase the complexity of the implantation [3]. Moreover, fully integrated coated stems are more difficult to revise.

Another result of our study was that component positioning primarily depends on preoperative intraarticular defect or extraarticular deformity given by mMPTA and mLDFA. John et al. presented three main concepts for managing complex knee deformities: 1. Primary TKA with symmetric or asymmetric bone resection, 2. Single-stage simultaneous corrective osteotomy and TKA, 3. Two-stage corrective osteotomy followed by delayed TKA [17]. However, most authors recommend intraarticular correction instead of simultaneous TKA and osteotomy due to technical difficulties and various complications (e.g. nonunion, stiffness, infection, fractures) [23]. Large extraarticular deformities cannot be addressed sufficiently using a non-modular arthroplasty system. The question arises as to whether reconstruction of the exact mechanical axis is absolutely necessary or whether a moderate deviation from neutral mechanical alignment can be accepted in hinged implants [25, 28]. However, recent developments in robotics and navigated knee arthroplasty offer the possibility to visualize implant positioning in real time intraoperatively. Extraarticular deformities can thus be addressed immediately by switching to a modular implant. Conclusions resulting from mMPTA and mLDFA measurements should take into account, that both values are resulting from intraarticular bone defects and extraarticular deformities, so that they might be pathologic even in cases without extraarticular deformity.

Both the incidence of periprosthetic fractures and the duration of surgical intervention were lower compared with the current literature, arguably due to the simplicity of the non-modular cementless system [19, 26]. Caron et al. determined 3% of periprosthetic fractures in their multicenter study of non-tumoral hinged knee arthroplasty [6]. Schnetz et al. reported 6% of periprosthetic fracture using modular cementless implants. Pour et al. and Hernandez-Vaquero who also investigated outcomes of modular systems identified rates of periprosthetic fractures of 2.3% and 3.8% [15, 24].

This study has several limitations. First it is an observational study, so that confounders cannot be controlled. There was no control group with cementless modular implants, so that fracture rates and operative time can only be compared with literature. Although the pre- and postoperative radiographs were performed in a standardized manner, projection errors by rotation of the leg and knee flexion limit the accuracy of the radiographic measurements.

## Conclusions

The main result of the present work is that a non-modular cemented rotating hinged knee arthroplasty system can reconstruct the mechanical leg axis precisely and comparable to modular cementless and conventional primary total knee arthroplasties. Potential malalignment is primarily dependent upon extraarticular deformity preoperatively. Based on these results we propose the use of either cemented or cementless modular implants with offset stem options in case of pathological mMPTA or mLDFA preoperatively. Periprosthetic fracture rates and duration of surgery were lower compared with current literature.

## Data Availability

The data that support the findings of this study are available on request from the corresponding author.

## Abbreviations

TKA: Total Knee Arthroplasty
RHK: Rotating Hinged Knee
ROM: Range Of Motion
mLDFA: Mechanical Lateral Distal Femoral Angle
mMPTA: Mechanical Medial Proximal Tibial Angle
resp.: Respectively
i.e.: Id est
e.g.: Exempli gratia
etc.: Et cetera
DICOM: Digital Imaging and Communications in Medicine
BMI: Body Mass Index
JLCA: Joint Line Convergent Angle
